# Some new soliton solutions of a semi-discrete fractional complex coupled dispersionless system

**DOI:** 10.1038/s41598-023-33689-9

**Published:** 2023-04-20

**Authors:** A. H. Abdel Kader, F. El Bialy, H. M. Nour, M. S. Abdel Latif

**Affiliations:** 1 Engineering Faculty, Mathematics and Engineering Physics Department, Mansoura University, Mansoura, Egypt; 2 Faculty of Science, Department of Mathematics, New Mansoura University, New Mansoura City, Egypt; 3 Basic Sciences Department, Mansoura Higher Institute for Engineering and Technology, Mansoura, Egypt

**Keywords:** Applied mathematics, Optical physics

## Abstract

In this paper, a semi-discrete fractional derivative complex coupled dispersionless system is proposed. The properties of M-fractional derivative are utilized to convert discrete M-fractional derivative system to a classical discrete differential system. Then the invariant subspace method (ISM) is utilized to find dark, bright, kink and W-shaped soliton solutions for the proposed system.

## Introduction

Dispersionless integrable equations have many valuable applications in physics and mathematics such as in quantum fields^1–6^. Coupled dispersionless (CD) system is firstly modeled by Konno and Oono^7^ and it was solved using the inverse scattering transform. After that, CD systems is developed to include generalized CD and three dimensions Euclidean space^7,8^. Several methods have been used to solve both of real CD equations^9–12^ and complex CD equations^13,14^. In Refs.^15,16^, real semi-discrete coupled dispersionless (SDCD) system is analyzed and soliton solutions are found.

In Ref.^17^, the bright soliton solution, breather solution and rogue wave solution of the semi-discrete complex coupled dispersionless integrable system (SDCCD) are obtained using Lax pairs and Darboux transformation method. Also, the modulational instability of SDCCD is studied in^20^.

The nonlinear phenomena have been accurately described by the M-fractional derivative. Such as in wave propagation, gravity wave propagation, optics, and in air pollutant dispersion^18,19^. So, in this paper, we introduce the semi-discrete M-fractional derivative complex coupled dispersionless (FDSDCCD) system

1a $${D}_{M}^{\alpha ,\beta }{q}_{n}+\left(\left|{R}_{n+1}\right|+|{R}_{n}|\right)\left(\left|{R}_{n+1}\right|-|{R}_{n}|\right)=0,$$

1b $${D}_{M}^{\alpha ,\beta }\left({R}_{n+1}-{R}_{n}\right)-\left({R}_{n+1}+{R}_{n}\right){q}_{n}=0,$$

where ${q}_{n}=q(n,t)$, ${R}_{n}=R(n,t)$ and ${R}_{n+1}=R(n+1,t)$. The FDSDCCD system physically used for describing the interaction of current-fed string in a certain external magnetic field^20^.

There are many methods proposed in the literature for obtaining exact solutions of differential-difference equations (D $\Delta$ Es). Examples of these methods are, Hirota method^21^, exponential function method^22,23^, square operator method^24,25^, similarity transformation method^26,27^, and neural network methods^28^. One of the effective methods for getting exact solution of D $\Delta$ Es such as Eqs. (1a) and (1b) is the ISM. It is successfully applied on partial differential equations and D $\Delta$ Es^29–32^.

In this paper, we use the ISM to obtain some novel exact soliton solutions of the system (1a) and (1b). The obtained soliton solutions are in the form of dark, bright, kink and W-shaped soliton.

This paper is organized as follows: In section “Some properties of the local M-derivative”, some properties of the Local M-Derivative are introduced. In section “The ISM”, the details of the ISM is explained. In section “Solutions of the system (1)”, some new exact soliton solutions are obtained using the ISM. In section “Conclusion”, we give the conclusion of this work.

## Some properties of the local M-derivative

The M-fractional derivative of $f(t)$ with order $\alpha$ (where $\alpha \in [\mathrm{0,1}]$) is defined as^18^

2 $${D}_{M}^{\alpha ,\beta }f\left(t\right)=\underset{\epsilon \to 0}{\mathit{lim}}\frac{f\left(t {E}_{\beta }\left(\epsilon {t}^{-\alpha }\right)\right)-f(t)}{\epsilon },$$

where

$${E}_{\beta }\left(x\right)=\sum_{j=0}^{\infty }\frac{{x}^{j}}{\Gamma (j\beta +1)},$$

is called the one-parameter Mitttag-Leffler function^18,19,32^. The M-fractional derivative has the same properties of classical derivatives in linearity, multiplication, and division of two functions but what distinguishes the M-fractional derivative are the following properties^18,19,32^:

$$\mathbf{i}.\,{D}_{M}^{\alpha ,\beta }f\left(g(t)\right)={f}^{^{\prime}}\left(g(t)\right){D}_{M}^{\alpha ,\beta }g(t),$$

$$\mathbf{i}\mathbf{i}.\,{D}_{M}^{\alpha ,\beta }f\left(\frac{\Gamma \left(\beta +1\right)}{\alpha }{t}^{\alpha }\right)=\frac{df(t)}{dt}.$$

## The ISM

In this section, we give a brief description of the ISM and how we can use it to get exact solutions of Eqs. (1a) and (1b).

Consider the following D $\Delta$ Es

3a $$\frac{d}{dt}{u}_{n}={F}_{1}\left[{u}_{n},{v}_{n},{\varphi }_{n}\right]={H}_{1}\left(n,{u}_{n},{{v}_{n},{\varphi }_{n},u}_{n+1},{v}_{n+1},{\varphi }_{n+1},\dots \right),$$

3b $$\frac{d}{dt}{v}_{n}={F}_{2}\left[{u}_{n},{v}_{n},{\varphi }_{n}\right]={H}_{2}\left(n,{u}_{n},{{v}_{n},{\varphi }_{n},u}_{n+1},{v}_{n+1},{\varphi }_{n+1},\dots \right),$$

3c $$\frac{d}{dt}{\varphi }_{n}={F}_{3}\left[{u}_{n},{v}_{n},{\varphi }_{n}\right]={H}_{3}\left(n,{u}_{n},{{v}_{n},{\varphi }_{n}, u}_{n+1},{v}_{n+1},{\varphi }_{n+1},\dots \right),$$

where ${u}_{n}=u\left(n,t\right)$, ${u}_{n+1}=u\left(n+1,t\right)$ … etc. To solve the Eqs. (3a), (3b), (3c) using the ISM, the following steps should be applied:

**Step 1**: Assume that the solution of Eqs. (3a), (3b), (3c) can be formulated as

4 $${u}_{n}=\sum_{i=1}^{k}{A}_{i}\left(t\right) \,{B}_{i}\left(n\right),\, {v}_{n}=\sum_{i=1}^{k}{C}_{i}\left(t\right) \,{D}_{i}\left(n\right), {\varphi }_{n}=\sum_{i=1}^{k}{L}_{i}\left(t\right)\, {J}_{i}\left(n\right),$$

where $k$ is a suitable selected dimension of the invariant subspace.

**Step 2**: The functions ${B}_{i}\left(n\right)$, ${D}_{i}\left(n\right)$ and ${J}_{i}\left(n\right)$ are the basis of the solution of the difference equations

5a $$\left({E}^{k}+{t}_{k-1} \,{E}^{k-1}+\dots +{t}_{1}\,E+{t}_{0}\right){y}_{1}(n)=0,$$

5b $$\left({E}^{k}+{r}_{k-1}\, {E}^{k-1}+\dots +{r}_{1}\,E+{r}_{0}\right){y}_{2}(n)=0,$$

5c $$\left({E}^{k}+{s}_{k-1}\, {E}^{k-1}+\dots +{s}_{1}\,E+{s}_{0}\right){y}_{3}(n)=0,$$

where $E$ is a difference shift $\left({E}^{k}y\left(n\right)=y\left(n+k\right) \right)$ and ${t}_{k-1},{r}_{k-1} ,\dots , {t}_{0}, {r}_{0}$ are constants which can be found by solving the determining equations that emerge from the following equations

6a $$\left({E}^{k}+{t}_{k-1} \,{E}^{k-1}+\dots +{t}_{1}E+{t}_{0}\right){{F}_{1}[y}_{1}\left(n\right),{y}_{2}\left(n\right),{y}_{3}(n)]=0,$$

6b $$\left({E}^{k}+{r}_{k-1} \,{E}^{k-1}+\dots +{r}_{1}E+{r}_{0}\right){{F}_{2}[y}_{1}\left(n\right),{y}_{2}\left(n\right),{y}_{3}(n)]=0.$$

6c $$\left({E}^{k}+{s}_{k-1} \,{E}^{k-1}+\dots +{s}_{1}E+{s}_{0}\right){{F}_{3}[y}_{1}\left(n\right),{y}_{2}\left(n\right),{y}_{3}(n)]=0.$$

**Step 3**: Solving the system of differential equations in ${A}_{i}\left(t\right)$, ${C}_{i}\left(t\right)$ and ${L}_{i}\left(t\right)$ which can be found by substituting Eq. (4) into Eqs. (3a), (3b), (3c) and equating the coefficients of the powers of ${y}_{1},{y}_{2},{y}_{3}$ and its first shift by zero.

## Solutions of the system (1a and 1b)

Consider the transformation

7 $${q}_{n}=q\left(n,\eta \right), \,{R}_{n}=r(n,\eta ).$$

where

8 $$\eta =\frac{\Gamma (\beta +1)}{\alpha }{t}^{\alpha }.$$

Appling properties of M-fractional derivative, system (1a and 1b) will be transformed into

9a $$\frac{d}{d\eta }{q}_{n}+\left(\left|{r}_{n+1}\right|+|{r}_{n}|\right)\left(\left|{r}_{n+1}\right|-|{r}_{n}|\right)=0,$$

9b $$\frac{d}{d\eta }\left({r}_{n+1}-{r}_{n}\right)-\left({r}_{n+1}+{r}_{n}\right){q}_{n}=0,$$

where ${q}_{n}=q\left(n,\eta \right),$ ${r}_{n}=r(n,\eta )$ and ${r}_{n+1}=r(n+1,\eta )$. Let

10a $${q}_{n}=Q\left(n,\eta \right)+i W\left(n\right),$$

10b $${r}_{n}=M\left(n,\eta \right)+i H\left(n,\eta \right).$$

Substituting Eqs. (10a), (10b) into Eqs. (9a), (9b) and separating real and imaginary parts, we find

11a $$\frac{d}{d\eta }{Q}_{n}={H}_{n}^{2}-{H}_{n+1}^{2}+{M}_{n}^{2}-{M}_{n+1}^{2} ,$$

11b $$\frac{d}{d\eta }{M}_{n}-\frac{d}{d\eta }{M}_{n+1}=-\left({M}_{n}+{M}_{n+1}\right){Q}_{n}+\left({H}_{n}+{H}_{n+1}\right){W}_{n} ,$$

11c $$\frac{d}{d\eta }{H}_{n}-\frac{d}{d\eta }{H}_{n+1}=-\left({H}_{n}+{H}_{n+1}\right){Q}_{n}-\left({M}_{n}+{M}_{n+1}\right){W}_{n} .$$

Using two-dimensional invariant subspace ($$k=2)$$, the solutions of the system (11a, 11b and 11c) can be expressed as

12a $$Q\left(n,\eta \right)={A}_{1}\left(\eta \right){D}_{1}\left(n\right)+{A}_{2}\left(\eta \right){D}_{2}\left(n\right),$$

12b $$M\left(n,\eta \right)={B}_{1}\left(\eta \right){S}_{1}\left(n\right)+{B}_{2}\left(\eta \right){S}_{2}\left(n\right),$$

12c $$H\left(n,\eta \right)={G}_{1}\left(\eta \right){N}_{1}\left(n\right)+{G}_{2}\left(\eta \right){N}_{2}\left(n\right),$$

where $\left\{{D}_{1},{D}_{2}\right\},\left\{{S}_{1},{S}_{2}\right\}, \{{N}_{1},{N}_{2}\}$ are the basic fundamental solutions of the following second order difference equations respectively

13a $${y}_{1}(n+2)+{t}_{1}{y}_{1}(n+1)+{t}_{0}{y}_{1}(n)=0,$$

13b $${y}_{2}(n+2)+{r}_{1}{y}_{2}(n+1)+{r}_{0}{y}_{2}(n)=0,$$

13c $${y}_{3}(n+2)+{s}_{1}{y}_{3}(n+1)+{s}_{0}{y}_{3}(n)=0.$$

The system (11a, 11b and 11c) can be rewritten in the form

14a $$\frac{d}{d\eta }{Q}_{n}={F}_{1}\left[{Q}_{n},{M}_{n},{H}_{n}\right]={H}_{n}^{2}-{H}_{n+1}^{2}+{M}_{n}^{2}-{M}_{n+1}^{2} ,$$

14b $$\frac{d}{d\eta }{M}_{n}-\frac{d}{d\eta }{M}_{n+1}={F}_{2}\left[{Q}_{n},{M}_{n},{H}_{n}\right]=-\left({M}_{n}+{M}_{n+1}\right){Q}_{n}+\left({H}_{n}+{H}_{n+1}\right){W}_{n} ,$$

14c $$\frac{d}{d\eta }{H}_{n}-\frac{d}{d\eta }{H}_{n+1}={F}_{3}\left[{Q}_{n},{M}_{n},{H}_{n}\right]=-\left({H}_{n}+{H}_{n+1}\right){Q}_{n}-\left({M}_{n}+{M}_{n+1}\right){W}_{n} .$$

Let,

15a $${F}_{1}[{y}_{1},{y}_{2},{y}_{3}]={{y}_{3}(n)}^{2}-{{y}_{3}\left(n+1\right)}^{2}+{{y}_{2}\left(n\right)}^{2}-{{y}_{2}\left(n+1\right)}^{2},$$

15b $${F}_{2}[{y}_{1},{y}_{2},{y}_{3}]=-\left({y}_{2}\left(n\right)+{y}_{2}\left(n+1\right)\right){y}_{1}\left(n\right)+\left({y}_{3}\left(n\right)+{y}_{3}\left(n+1\right)\right){W}_{n},$$

15c $${F}_{3}[{y}_{1},{y}_{2},{y}_{3}]=-\left({y}_{3}\left(n\right)+{y}_{3}\left(n+1\right)\right){y}_{1}\left(n\right)-\left({y}_{2}\left(n\right)+{y}_{2}\left(n+1\right)\right){W}_{n}.$$

Substituting Eqs. (13a), (13b), (13c) and Eqs. (15a), (15b), (15c) into Eqs. (6a), (6b), (6c) and solving the obtained determining equations, we get

16 $${t}_{0}=-1, \,{t}_{1}=0, \,{r}_{0}=-1, \,{r}_{1}=0, \,{s}_{0}=-1, \,{s}_{1}=0,$$

17 $${W}_{n}={\alpha }_{1}+{(-1)}^{n}{\alpha }_{2},$$

where ${\alpha }_{1}$ and ${\alpha }_{2}$ are constants. Substituting Eq. (16) into Eqs. (13a), (13b), (13c) and solving Eqs. (13a), (13b), (13c), we find

18a $${y}_{1}\left(n\right)={a}_{1}+{(-1)}^{n}{a}_{2},$$

18b $${y}_{2}\left(n\right)={a}_{3}+{(-1)}^{n}{a}_{4},$$

18c $${y}_{3}\left(n\right)={a}_{5}+{(-1)}^{n}{a}_{6},$$

where ${a}_{1},{a}_{2}, \dots , {a}_{6}$ are constants. So the solutions of Eqs. (11a), (11b), (11c) take the form

19a $${Q}_{n}={\left(-1\right)}^{n}{A}_{1}\left(\eta \right)+{A}_{2}\left(\eta \right),$$

19b $${M}_{n}={\left(-1\right)}^{n}{B}_{1}\left(\eta \right)+{B}_{2}\left(\eta \right),$$

19c $${H}_{n}={\left(-1\right)}^{n}{G}_{1}\left(\eta \right)+{G}_{2}\left(\eta \right).$$

Substituting Eqs. (19a), (19b), (19c) and Eq. (17) into Eqs. (11a), (11b), (11c), we find

20a $${(-1)}^{n}\left(4{B}_{1}{B}_{2}+4{G}_{1}{G}_{2}-{{A}_{1}}^{^{\prime}}\right)={{A}_{2}}^{^{\prime}},$$

20b $${\left(-1\right)}^{n}\left({A}_{1}{B}_{2}+{{B}_{1}}^{^{\prime}}-{\alpha }_{2}{G}_{2}\right)+{A}_{2}{B}_{2}-{\alpha }_{1}{G}_{2}=0,$$

20c $${\alpha }_{1}{B}_{2}+{A}_{2}{G}_{2}+{(-1)}^{n}\left({\alpha }_{2}{B}_{2}+{A}_{1}{G}_{2}+{{G}_{1}}^{^{\prime}}\right)=0.$$

Comparing the coefficient of ${(-1)}^{n}$ in Eq. (20a), we find

21 $${A}_{2}={\alpha }_{3},$$

Substituting Eq. (21) into Eqs. (20b) and (20c) and then comparing the coefficient of ${(-1)}^{n}$ in Eqs. (20b) and (20c), we find

22 $${\alpha }_{1}={\alpha }_{3}=0.$$

Substituting Eqs. (21) and (22) into Eq. (20b), then solving Eq. (20b), we find

23 $${B}_{2}=\frac{{\alpha }_{2}{G}_{2}-{{B}_{1}}^{^{\prime}}}{{A}_{1}}.$$

Substituting Eq. (23) into Eq. (20c), we find

24 $${G}_{2}=\frac{{\alpha }_{2}{{B}_{1}}^{^{\prime}}-{A}_{1}{{G}_{1}}^{^{\prime}}}{{\alpha }_{2}^{2}+{{A}_{1}}^{2}}.$$

Substituting Eqs. (23) and (24) into Eq. (20a), we find

25 $${{A}_{1}}^{^{\prime}}\left({\alpha }_{2}^{2}+{{A}_{1}}^{2}\right)+4\left({\alpha }_{2}\left(-{G}_{1}{{B}_{1}}^{^{\prime}}+{B}_{1}{{G}_{1}}^{^{\prime}}\right)+{A}_{1}({B}_{1}{{B}_{1}}^{^{\prime}}+{G}_{1}{{G}_{1}}^{^{\prime}})\right)=0.$$

To Solve Eq. (25), we distinguish the following two cases:

**Case 1**: The first solution of Eq. (25) is given by

26 $${A}_{1}\left(\eta \right)=2\frac{{c}_{2}\sqrt{{c}_{4}}\mathrm{tanh}\left(\sqrt{{c}_{4}}\left(\eta +{c}_{3}\right)\right)}{{c}_{1}},$$

27 $${B}_{1}\left(\eta \right)={c}_{5} {\mathrm{tan}}^{-1}\left(\mathrm{sinh}\left(\sqrt{{c}_{4}}\left(\eta +{c}_{3}\right)\right)\right)+{c}_{6},$$

28 $${\alpha }_{2}=-2{c}_{5},$$

29 $${G}_{1}=-\frac{{c}_{2}\sqrt{{c}_{4}}}{{c}_{1}}\mathrm{sech}\left(\sqrt{{c}_{4}}\left(\eta +{c}_{3}\right)\right).$$

where ${c}_{1}, {c}_{2}, \dots , {c}_{5}$ are constants. In this case, the solution of Eqs. (9a), (9b) is

30 $${r}_{n}={\left(-1\right)}^{n}\left({c}_{5}{tan}^{-1}\left(\mathit{sin}h\left(\sqrt{{c}_{4}}\left({c}_{3}+\eta \right)\right)\right)+{c}_{6}\right)-i\left(\frac{\sqrt{{c}_{4}}}{2}+\frac{{c}_{2}}{{c}_{1}}\sqrt{{c}_{4}} {\left(-1\right)}^{n}\right)\mathit{sec}h\left(\sqrt{{c}_{4}}\left({c}_{3}+\eta \right)\right),$$

31 $${q}_{n}=2{\left(-1\right)}^{n}\left(-i{c}_{5}+\frac{\sqrt{{c}_{4}}{c}_{2}}{{c}_{1}}\mathit{tan}h\left(\sqrt{{c}_{4}}\left({c}_{3}+\eta \right)\right)\right).$$

Substituting Eqs. (30) and (31) into Eq. (7) taking into the consideration Eq. (8), then taking the absolute value of Eq. (7), we find

32 $${|q}_{n}|=\sqrt{4{c}_{5}^{2}+\frac{{4c}_{4}{c}_{2}^{2}}{{c}_{1}^{2}}{\mathit{tan}h}^{2}\left(\sqrt{{c}_{4}}\left({c}_{3}+\frac{\Gamma \left(\beta +1\right)}{\alpha }{t}^{\alpha }\right)\right)},$$

33 $${\left|{R}_{n}\right|}^{2}={\left({c}_{5}{tan}^{-1}\left(\mathit{sin}h\left(\sqrt{{c}_{4}}\left({c}_{3}+\frac{\Gamma \left(\beta +1\right)}{\alpha }{t}^{\alpha }\right)\right)\right)+{c}_{6}\right)}^{2}+{\left(\frac{\sqrt{{c}_{4}}}{2}+\frac{{c}_{2}}{{c}_{1}}\sqrt{{c}_{4}} {\left(-1\right)}^{n}\right)}^{2}{\mathit{sec}h}^{2}\left(\sqrt{{c}_{4}}\left({c}_{3}+\frac{\Gamma \left(\beta +1\right)}{\alpha }{t}^{\alpha }\right)\right).$$

The arbitrary parameters in Eqs. (32) and (33) can be chosen to distinguish many physical solutions such as bright, dark and W-shaped soliton solutions as illustrated in the following figures.

**Figure 1:**
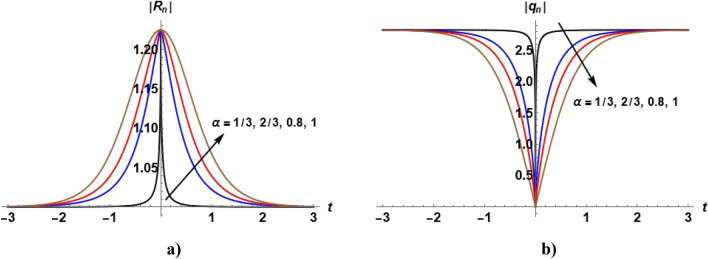
Plot of the solutions (32) and (33) when ${c}_{1}={c}_{2}={c}_{6}=1, {c}_{3}={c}_{5}=0$ $,{c}_{4}=2, \beta =0.5$ and odd values of $n$ for different values of $\alpha$.

**Figure 2:**
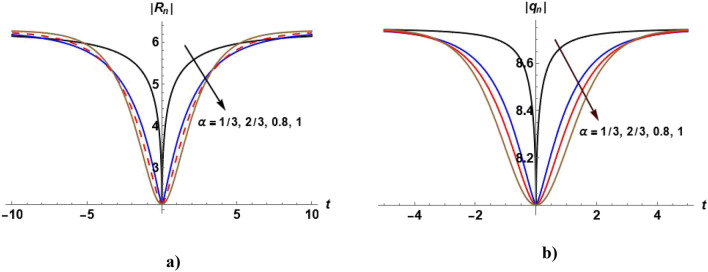
Plot of the solutions (32) and (33) when ${c}_{1}=-2,{c}_{2}=5,{c}_{5}=4, {c}_{3}={c}_{6}=0$ $,{c}_{4}=\beta =0.5$ and odd values of $n$ for different values of $\alpha$.

**Figure 3:**
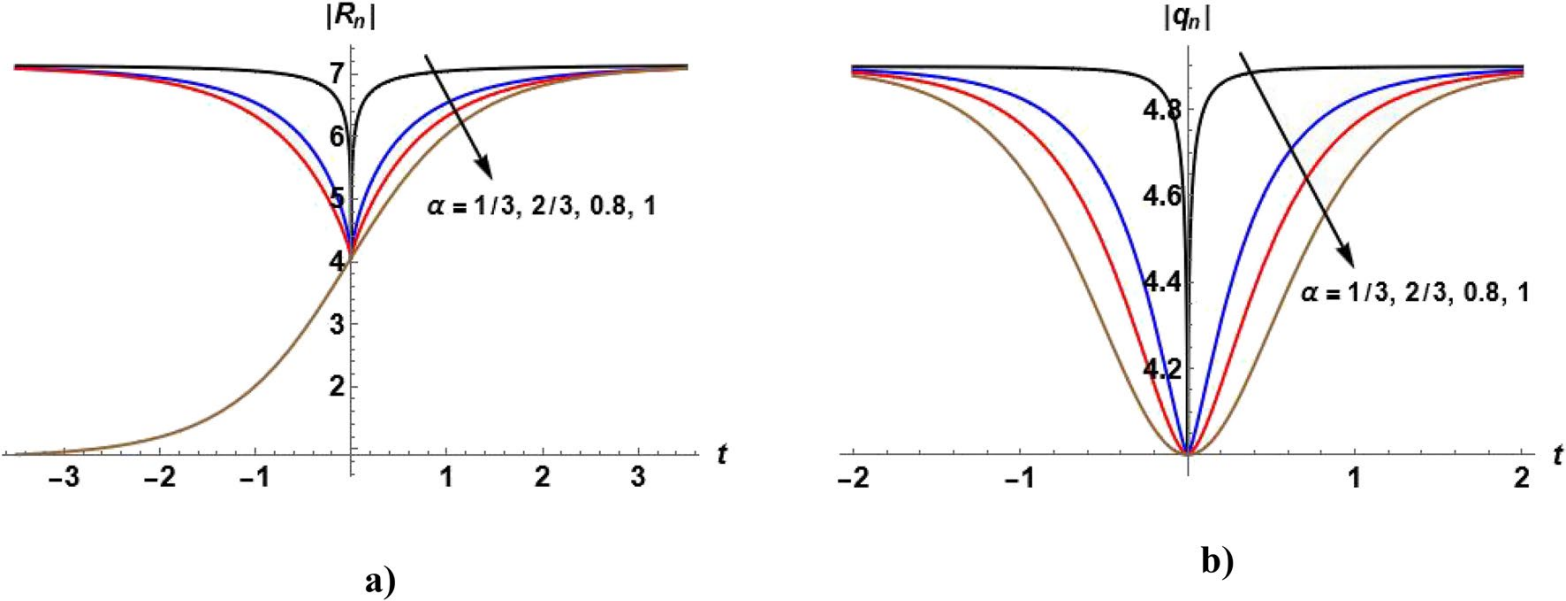
Plot of the solutions (32) and (33) when ${c}_{1}={c}_{2}=1,{{c}_{4}=c}_{5}=2, {c}_{3}=0,{c}_{6}=4$ $,\beta =0.5$ and odd values of $n$ for different values of $\alpha$.

**Figure 4:**
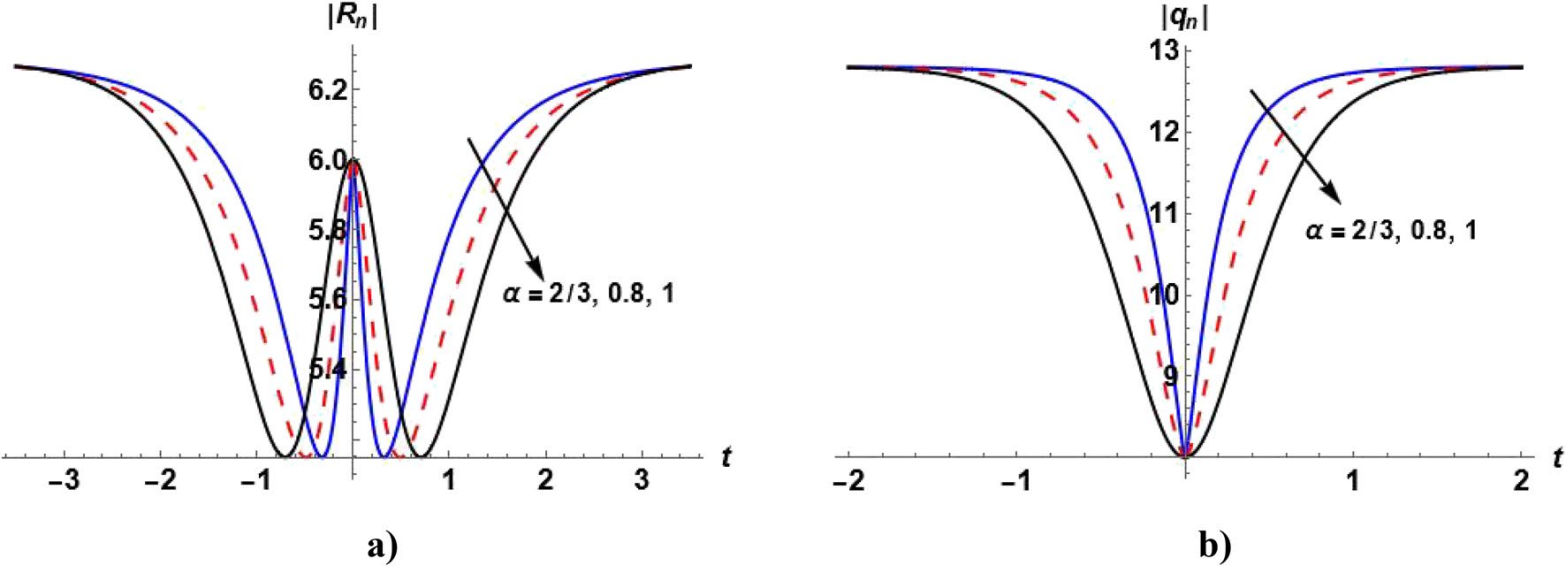
Plot of the solutions (32) and (33) when ${c}_{1}=-2,{c}_{2}=5,{{c}_{4}=c}_{5}=4, {c}_{3}={c}_{6}=0$ $,\beta =0.5$ and odd values of $n$ for different values of $\alpha$.

**Figure 5:**
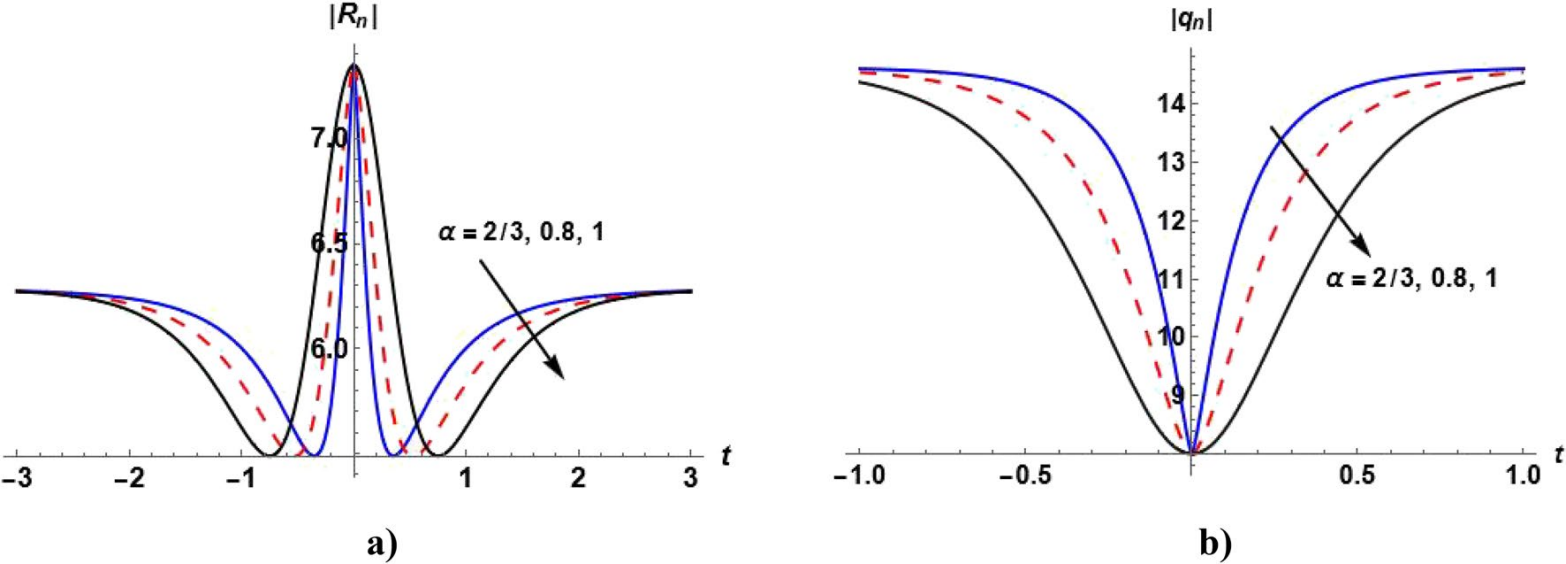
Plot of the solutions (32) and (33) when ${c}_{1}=-2,{c}_{2}=5,{{c}_{4}=6,c}_{5}=4, {c}_{3}={c}_{6}=0$ $,\beta =0.5$ and odd values of $n$ for different values of $\alpha .$

Figures 1, 2, 3, 4 and 5 show the effect of the parameter $\alpha$ on the profile of $\left|{R}_{n}\right|$ and $\left|{q}_{n}\right|$. Figure 1a shows the bright soliton solution (30) in which we can notice that with increasing the value of $\alpha ,$ the value of $\left|{R}_{n}\right|$ increases with time. Figure 2a shows the dark soliton of the solution (30) and we notice that with increasing the value of $\alpha ,$ the value of $\left|{R}_{n}\right|$ decreases with time. Figure 3a show that when $\alpha =1,$ the kink soliton of the solution (30) is obtained and with decreasing the value of $\alpha ,$ the profile of ${R}_{n}$ transforms to dark soliton. Figure 4a and Fig. 5(a) show the W-shaped soliton of the solution (30) and with increasing the value of $\alpha ,$ the value of $\left|{R}_{n}\right|$ decreases with time. Figures 1b, 2b, 3b, 4b and 5b show the dark soliton solution (31) and with increasing the value of $\alpha ,$ the value of ${q}_{n}$ decreases with time. The parameter $\alpha$ can be used to control the shape of the solution which can make it more realistic.

**Case 2**: Let

34 $${B}_{1}(\eta )={G}_{1}\left(\eta \right).$$

Substituting Eq. (34) into Eq. (25), we find

35 $$\left({\alpha }_{2}^{2}+{A}_{1}^{2}\right){A}_{1}^{^{\prime}}=-8{A}_{1}{G}_{1}{{G}_{1}}^{^{\prime}}.$$

Solving Eq. (35), we find

36 $${G}_{1}\left(\eta \right)=\frac{\sqrt{16{\alpha }_{4}-2 {\alpha }_{2}^{2}\mathrm{ln}\left({A}_{1}(\eta )\right)-{{A}_{1}(\eta )}^{2}}}{2\sqrt{2}},$$

where ${\alpha }_{4}$ is a constant of integration. Then the solution of Eqs. (9a), (9b) is in terms of an arbitrary function ${A}_{1}(\eta )$ as follows

37 $${r}_{n}=\left(\frac{1}{2}+\frac{i}{2}\right)\frac{-{\left(-1\right)}^{n}{A}_{1}^{3}-i{\alpha }_{2}{A}_{1}^{^{\prime}}+{A}_{1}\left({\left(-1\right)}^{n}{\alpha }_{4}-2{\left(-1\right)}^{n}{\alpha }_{2}^{2}\mathit{ln}\left({A}_{1}\right)+{A}_{1}^{^{\prime}}\right)}{\sqrt{2} {A}_{1}\sqrt{{\alpha }_{4}-2{\alpha }_{2}^{2}\mathit{ln}\left({A}_{1}\right)-{A}_{1}^{2}}},$$

38 $${q}_{n}={(-1)}^{n}(i{\alpha }_{2}+{A}_{1}).$$

Substituting Eq. (37) and Eq. (38) into Eq. (7) with taking into the consideration Eq. (8), then taking the absolute value of Eq. (7), we find

39 $${|q}_{n}|=\sqrt{{\alpha }_{2}^{2}+{A}_{1}^{2}} ,$$

40 $${\left|{R}_{n}\right|}^{2}=\frac{1}{4 {{A}_{1}}^{2}\left({\alpha }_{4}-2{\alpha }_{2}^{2}\mathit{ln}\left({A}_{1}\right)-{A}_{1}^{2}\right)}\left({\alpha }_{2}^{2}{\left({A}_{1}^{^{\prime}}\right)}^{2}+{A}_{1}^{2}{\left({{A}_{1}^{^{\prime}}+(-1)}^{n}\left({\alpha }_{4}-2{\alpha }_{2}^{2}\mathit{ln}\left({A}_{1}\right)-{A}_{1}^{2}\right)\right)}^{2}\right),$$

where ${A}_{1}$ is a function in $\frac{\Gamma \left(\beta +1\right)}{\alpha }{t}^{\alpha }$.

**Figure 6:**
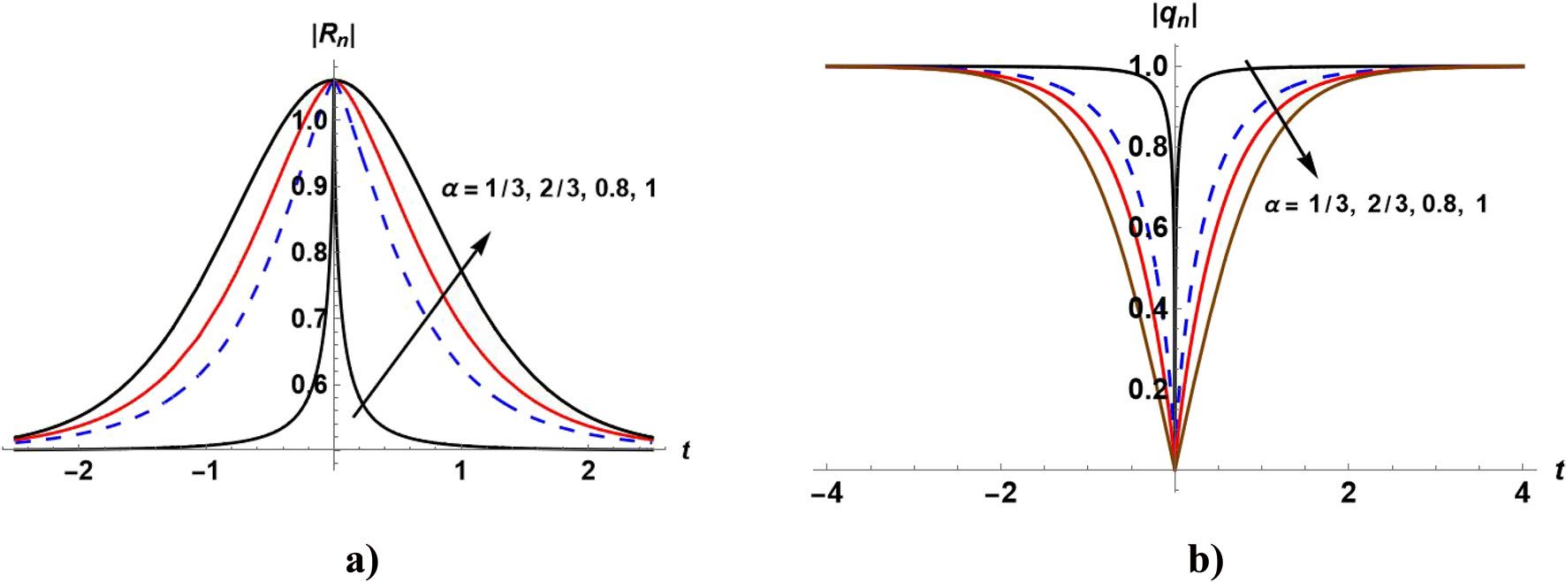
Plot of the solutions (39) and (40) when ${A}_{1}=\mathrm{tanh}\eta ,$ ${\alpha }_{4}=2,{\alpha }_{2}=0,$ $,\beta =1$ and even values of $n$ for different values of $\alpha .$

Figure 6a shows the bright soliton of the solution (40). With increasing the value of $\alpha ,$ the value of $\left|{R}_{n}\right|$ increases with time. Figure 6b shows the dark soliton solution (39). With increasing the value of $\alpha ,$ the value of $\left|{q}_{n}\right|$ decreases with time.

## Conclusion

In this paper, we used the properties of M-derivative to convert FDSDCCD system (1a and 1b) to SDCCD system (9a and 9b). After that, we applied the ISM to find some solutions for system (9a and 9b). The absolute values of the solution of FDSDCCD system Eqs. (30) and (31) are obtained. Numerical examples are introduced to explore various types of soliton solutions of the system (1a and 1b) such as dark, bright, kink and W-shaped solitons. The effect of the parameter $\alpha$ on the profile of the solutions is discussed.

## Data Availability

The datasets used and/or analyzed during the current study are available from the corresponding author upon reasonable request.
